# siRNA against presenilin 1 (PS1) down regulates amyloid β42 production in IMR-32 cells

**DOI:** 10.1186/1423-0127-19-2

**Published:** 2012-01-03

**Authors:** Ramesh JL Kandimalla, Willayat Yousuf Wani, Binukumar BK, Kiran Dip Gill

**Affiliations:** 1 Department of Biochemistry, Post Graduate Institute of Medical Education and Research, Chandigarh, India

**Keywords:** Alzheimer's Disease, presenilins, siRNA, Aβ42, IMR-32 Cells

## Abstract

**Background:**

One of the pathological hallmarks of Alzheimer's disease (AD) is the deposition of the ~4 kDa amyloid β protein (Aβ) within lesions known as senile plaques. Aβ is also deposited in the walls of cerebral blood vessels in many cases of AD. A substantial proportion of the Aβ that accumulates in the AD brain is deposited as Amyloid, which is highly insoluble, proteinaceous material with a β-pleated-sheet conformation and deposited extracellularly in the form of 5-10 nm wide straight fibrils. As γ-secretase catalyzes the final cleavage that releases the Aβ42 or 40 from amyloid β -protein precursor (APP), therefore, it is a potential therapeutic target for the treatment of AD. γ-Secretase cleavage is performed by a high molecular weight protein complex containing presenilins (PSs), nicastrin, Aph-1 and Pen-2. Previous studies have demonstrated that the presenilins (PS1 and PS2) are critical components of a large enzyme complex that performs γ-secretase cleavage.

**Methods:**

In this study we used RNA interference (RNAi) technology to examine the effects of small-interfering RNA (siRNA) against PS1 on expression levels of PS1 and Aβ42 in IMR-32 Cells using RTPCR, western blotting and immunofluorescence techniques.

**Results:**

The results of the present study showed down regulation of PS1 and Aβ42 in IMR32 cells transfected with siRNA against PS1.

**Conclusion:**

Our results substantiate the concept that PS1 is involved in γ-secretase activity and provides the rationale for therapeutic strategies aimed at influencing Aβ42 production.

## Background

A key step in the pathogenesis of AD is proteolysis of APP that results in the formation of the amyloid-β protein (Aβ), the principle protein component of the characteristic cerebral plaques of the disease [1]. Aβ is produced from APP ﬁrst by the action of β- secretase, a membrane-tethered enzyme that resembles pepsin and other water-soluble aspartyl proteases [2]. This proteolysis leads to membrane shedding of the large luminal/extracellular APP domain. The 99-residue membrane-bound remnant is then cleaved in the middle of its transmembrane region by γ -secretase, releasing Aβ and again near the inner leaﬂet at the β site to release the APP intracellular domain (AICD) [3]. Rare mutations in the APP gene, found in and around the Aβ region cause familial early-onset Alzheimer's disease (EOAD) and these mutations alter the production of Aβ or its aggregation properties [4]. Several contemporaneous observations provided critical clues for the identiﬁcation of the elusive γ-secretase. First, genes encoding the multi-pass membrane proteins presenilin-1 and presenilin-2 (PS1&PS2) were discovered in a search to identify other genes associated with familial EOAD. The disease-causing missense mutations were soon found to alter how γ -secretase cuts APP leading to increased proportions of longer, more aggregation-prone forms of Aβ [5]. Second, knock out of PS-1 dramatically reduced γ -secretase cleavage of APP [6]. Third, the types of compounds that could inhibit γ -secretase contained moieties typically found in aspartyl protease inhibitors [7]. These ﬁndings led to the identiﬁcation of two conserved transmembrane aspartates in the multi-pass presenilin that are critical for γ -secretase cleavage of APP, suggesting that presenilins might be the responsible aspartyl proteases [8]. Presenilin is cut into two pieces, an N-terminal fragment (NTF) and a C-terminal fragment (CTF), the formation of which is gated by limiting cellular factor(s) [9]. NTF and CTF remain physically associated in a high molecular weight complex and are metabolically stable [10]. These and other results suggested that the NTF-CTF heterodimer is the biologically active form [11,12]. The NTF and CTF each contributes one of the essential and conserved aspartates, suggesting that the γ -secretase active site might be at the interface between these two presenilin fragments. In strong support of this hypothesis, transition-state analogue inhibitors of γ -secretase, designed to interact with the active site of the protease, bind directly to presenilin NTF and CTF [13,14]. However, presenilin alone is not proteolytically active. This fact, along with the requirement for other factors for presenilin NTF/CTF formation and the assembly of presenilin into large complexes, suggested that γ- secretase is composed of other subunits besides presenilins. Further, Presenilin 1 (PS1) knockout mice and inhibitor cross-linking studies have provided convincing evidence that PS1 is an essential subunit for γ-secretase processing [15]. Recently, small interfering RNA (siRNA) has been found to be important in regulating protein expression in organisms varying from plants to mammals [16]. By activating a sequence-specific RNA degradation process, siRNA post transcriptionally inhibits protein expression of the specific gene [17,18]. We exploited siRNA technology to understand the regulation of γ-secretase by presenilin-1 and its impact on Aβ42 production in IMR-32 neuronal cell line. The results showed that there was PS1 down regulation and lesser Aβ42 production, suggesting PS1 is involved in γ-secretase activity and provides the rationale for therapeutic strategies aimed at influencing Aβ production.

## Methods

### 2.1 IMR-32 cell culture

Human neuroblastoma IMR-32 cells were obtained from NCCS, Pune, India and maintained at 37°C in an atmosphere of 95% air and 5% CO_2 _in Eagle's minimum essential medium supplemented with 2 mM L-glutamine and Earle's BSS, 1.5 g/L bicarbonate, 0.1 mM nonessential amino acids, 1.0 mM Sodium pyruvate and 10% heat-inactivated fetal bovine serum. Media was changed every two days. Passage was done in 1:3 ratios and doubling time was 24 Hours.

### 2.2 siRNA transfection against PS1

In a six well tissue culture plate, 2 × 10^5 ^cells were seeded per well in 2 ml antibiotic-free normal growth medium supplemented with FBS. The cells were incubated at 37°C in a CO_2 _incubator until the cells were 60-80% confluent for 18-24 hours. Transfection of PS1 siRNA (50 **pmole**s, 75 **pmoles **and 100 **pmoles **siRNA) and control siRNA duplex was done with the transfection reagent after checking the cell viability with the trypan blue and the cells were incubated for 5-7 hours at 37°C in a CO_2 _incubator. Then 1 ml of normal growth medium was added, containing 2 times the normal serum and antibiotics concentration (2× normal growth medium) without removing the transfection mixture and the cells were incubated for an additional 18-24 hours. After incubation, media was aspirated and replaced with 1X normal growth medium and incubated for 48 hours. After 48 hours cells were used for RNA (Taurus Scientific) and protein isolation, used for RT-PCR and western blotting analysis respectively.

### 2.3 Semi-Quantitative PCR amplification

Expression of PS1 after transfecting IMR-32 cells with siRNA against PS1 was evaluated by PCR analysis using sequence specific primers corresponding to the sequence in the open reading frame. 10 μl PCR mixture was prepared in 1× PCR buffer consisting of 1 unit of Taq polymerase, 2 μM of each primer for GAPDH and PS1 along with 200 μM of each dNTP. For amplification of PS 1 and GAPDH, cDNA products (1 μl) were subjected to reverse transcriptase PCR analysis on a gradient thermal cycler instrument (Eppendorff, Germany). PCR cycle for PS1 and control siRNA comprised of initial denaturation at 94°C for 2 min. The amplification was then carried out for 35 cycles consisting 45 sec each for 94°C (denaturation), 62°C (annealing) and 72°C, 1 min (elongation). Final extension was done at 72°C for 10 min. PCR cycle of GAPDH, which was used as internal control, comprised of initial denaturation at 94°C for 2 min. The amplification was then carried out for 35 cycles consisting 45 sec each for 94°C (denaturation), 56.8°C (annealing) and 72°C, 1 min (elongation). Final extension was done at 72°C for 10 min. Densitometric analysis of the products was done using SCION IMAGE software (Scion Image Corporation, Fredrick, MD, USA) to compare the relative mRNA expression of PS1 gene at various concentrations of PS1 siRNA duplex.

### 2.4 Immunoblotting

#### Immuno detection of PS 1

Following SDS-PAGE (12%), proteins were transferred to NC membrane with semi dry apparatus (Bio Rad) using transfer buffer [25 mM Tris, 192 mM glycine, 20% (vol/vol) methanol, pH 8.3]. The blots were incubated with 5% non fat dried milk for at least 2 h and then washed three times for 10 min each with PBS plus 0.1% Tween-20. Blots were incubated for 2.5 h at room temperature with anti PS1antibody (diluted 1:250 in PBS) (Santa cruz Biotechnology, Inc, USA). The primary antibody was removed, and the blots were washed three times for 10 min each with PBS plus 0.1% Tween-20. The blots were then incubated in horseradish peroxidase-labeled goat anti-rabbit IgG (diluted 1:5,000 in PBS) for 45 min at room temperature. After washing the membrane for at least 1 hour, the proteins were visualized by diaminobenzidine (DAB) from Bangalore Genei, India The densitometric analysis of the protein bands was carried out using SCION IMAGE software (Scion Image Corporation, Fredrick, MD, USA) to compare the relative expression of proteins.

#### Immuno detection of *Aβ42*

Following SDS-PAGE (**16**%), proteins were transferred to NC membrane with semi dry apparatus (Bio Rad) using transfer buffer [25 mM Tris, 192 mM glycine, 20% (vol/vol) methanol, pH 8.3]. The blots were incubated with 5% non fat dried milk for at least 2 h and then washed three times for 10 min each with PBS plus 0.1% Tween-20. Blots were incubated for 2.5 h at room temperature with anti Aβ42 antibody (diluted 1:250 in PBS) (Santa cruz Biotechnology, Inc, USA). The primary antibody was removed, and the blots were washed three times for 10 min each with PBS plus 0.1% Tween-20. The blots were then incubated in horseradish peroxidase-labeled goat anti-rabbit IgG (diluted 1:5,000 in PBS) for 45 min at room temperature. After washing the membrane for at least 1 hour, the proteins were visualized by diaminobenzidine (DAB), from Bangalore Genei, India. The densitometric analysis of the protein bands was carried out using SCION IMAGE software (Scion Image Corporation, Fredrick, MD, USA) to compare the relative expression of proteins.

#### Immunofluorescence staining of PS1 and Aβ42

IMR-32 cells were transfected with PS1 siRNA as described earlier and cells were fixed with the 1:1 Acetone/Methanol fixative for 20 minutes at 20°C. After 20 minutes fixative was aspirated and washed three times with the 1X PBS and proceeded for immunostaining. For immunostaining, fixed cells were blocked in the blocking buffer for 60 minutes at 37°C. After 60 minutes blocking buffer was aspirated and incubated with the anti PS1, anti Aβ42 (1:50 dilutions) (Santa cruz Biotechnology, Inc, USA) for 24 hours at 4°C. After 24 hours incubation, cells were rinsed three times with 1X PBS and incubated with FITC labelled secondary antibody (1:50 dilutions) for 2 hours in dark at 37°C. The cells were visualized under the fluorescence microscope and the images were recorded.

## Results

### 3.1 Effect of siRNA on the expression of presenilin 1 (PS1) gene in IMR-32 cells determined by RT-PCR

**Figure 1:**
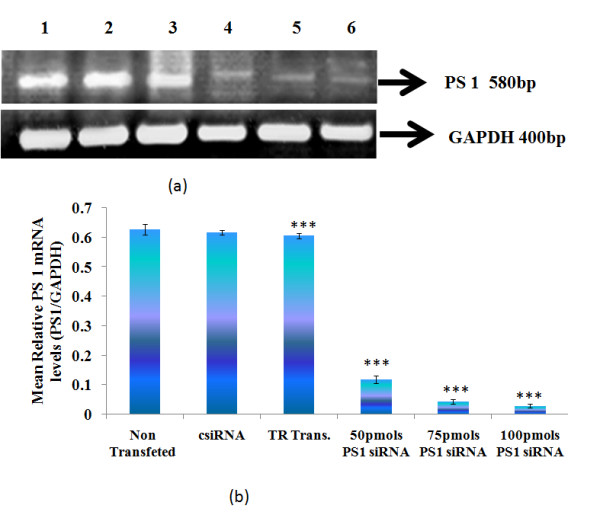
**RT-PCR analysis of PS1 (a) agarose gel electrophoresis and (b) densitometric analysis**. Values are mean ± SD (N = 5) Lane 1: Non siRNA transfected IMR-32 cells; Lane 2: Control siRNA transfected IMR-32 cells; Lane 3: Transfection Reagent transfected IMR-32 cells; Lane 4: 50 pmoles PS1 siRNA transfected IMR-32 cells; Lane 4: 75 pmoles PS1 siRNA transfected IMR-32 cells; Lane 5: 100 pmoles PS1 siRNA transfected IMR-32 cells. Values are mean ± SD (N = 5). *** p < 0.001 significantly different from csiRNA.

After attaining 60-80% confluency, IMR-32 cells were transfected with varying concentrations (50 **pmoles**, 75 **pmoles**, and 100 **pmoles**) of PS1 siRNA and 100 **pmoles **of control or scrambled siRNA. Semiquantitative PCR analysis of PS1 showed reduction in PS1mRNA in IMR-32 cells with increasing concentration of PS1 siRNA with maximum reduction at 100 **pmoles **(Figure 1).

### 3.2 Effect of siRNA on the content of PS1 peptide in IMR-32 cells

**Figure 2:**
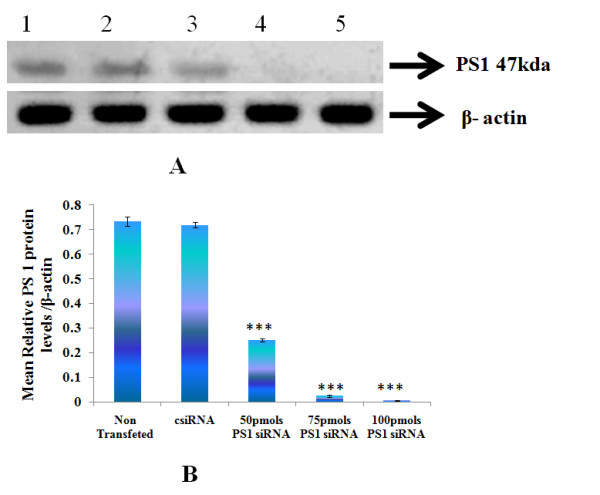
**Effect of PS1 siRNA on the protein levels of PS1 (a) Immunoblot analysis of PS1 (b) densitometric analysis**. 60 μg proteins from IMR 32 cells after PS1 transfection at different concentrations were subjected to westren blot analysis using an anti PS1 antibody (1:250 dilutions). Lane 1: Non siRNA Transfected IMR-32 Cells; Lane 2: Control siRNA; Lane 3: 50 pmoles PS1siRNA; Lane 4: 75 pmoles PS1 siRNA; Lane 5: 100 pmoles PS1 siRNA. Values are mean ± SD (N = 5). *** p < 0.001 significantly different from csiRNA.

Western blotting analysis of PS1 showed a decreased protein expression with increase in concentration of PS1 siRNA. In other words there was a dose dependent decrease in PS1 protein levels with increased concentration of PS1 siRNA(Figure 2).

### 3.4 Effect of PS1 siRNA on *Aβ42 *production in IMR-32 cells

**Figure 3:**
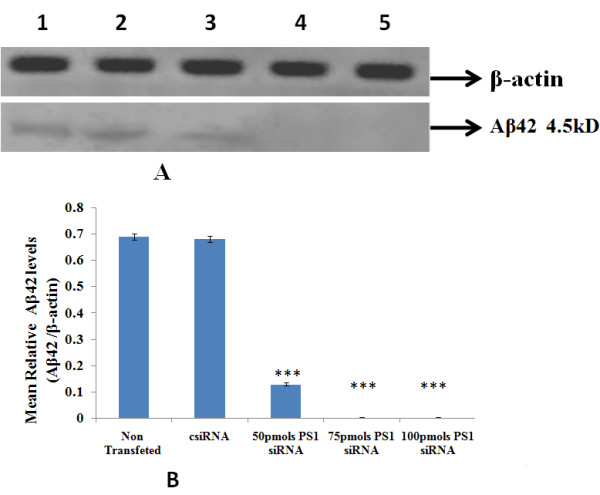
**Effect of PS1 siRNA on the protein levels Aβ42 (a) Immunoblot analysis of Aβ42 (b) densitometric analysis**. 60 μg proteins of IMR 32 cells after PS1 transfection at different concentrations were subjected to western blot analysis using anti Aβ42 antibody (1:250 dilutions). Lane 1: Non siRNA Transfected IMR-32 Cells; Lane 2: Control siRNA; Lane 3: 50 pmoles PS1siRNA; Lane 4: 75 pmoles PS1 siRNA; Lane 5:100 pmoles PS1 siRNA. Values are mean ± SD (N = 5). *** p < 0.001 significantly different from csiRNA.

Aβ42 expression was studies were carried out using western blotting, after IMR32 cells were transfected with the PS1 siRNA, 50 **pmoles **PS1 siRNA was able to reduce the Aβ42 expression, but at 75 **pmoles **and 100 **pmoles **of PS1 siRNA the expression was completely reduced (Figure 3).

### 3.5 Immunofuorescence analysis of PS1 and Aβ42

**Figure 4:**
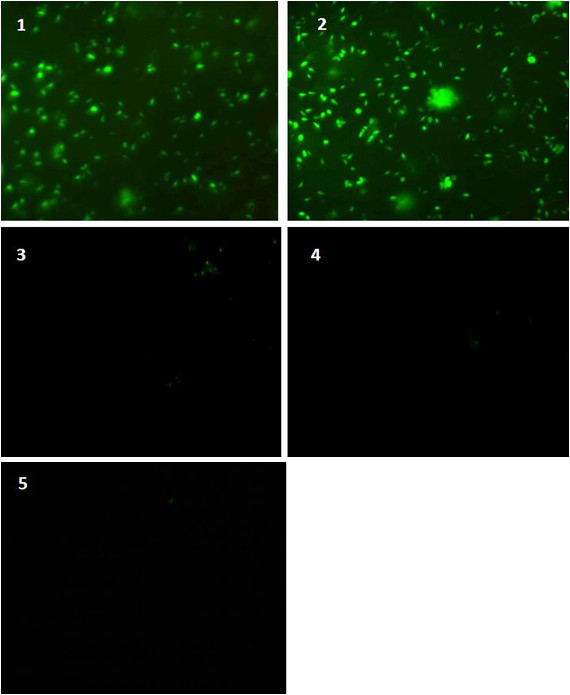
**Immunofluorescence analysis of Aβ42 in methanol acetone fixed IMR-32 cells after transfected with PS1 siRNA and control siRNA**. Plane 1: Non siRNA transfected IMR-32 Cells; Plane 2: Control siRNA; Plane 3: 50 pmoles PS1 siRNA; Plane 4: 75 pmoles PS1 siRNA; Plane 5: 100 pmols PS1 siRNA. PS1 siRNA and control siRNA transfected IMR-32 cells were probed with Aβ42 antibody. PS1 siRNA and control siRNA transfected IMR-32 cells were probed with anti Aβ42 antibody. The signal was revealed using FITC-conjugated secondary antibody and visualized on a fluorescence microscope (o.m. 10X).

**Figure 5:**
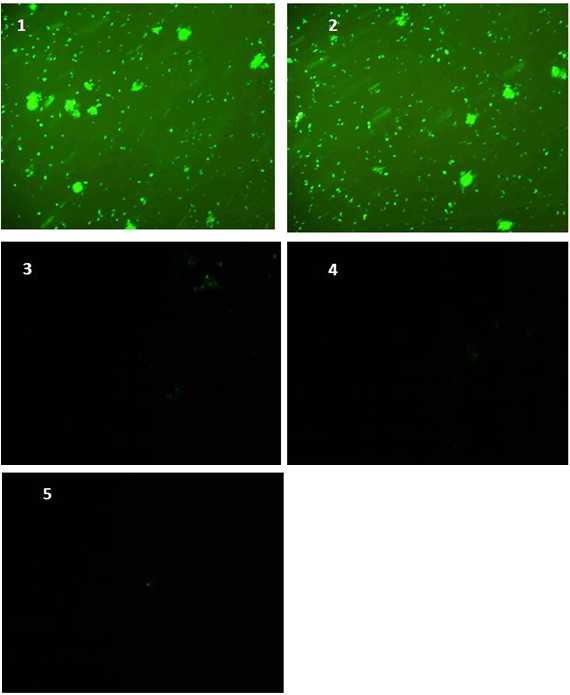
**Immunofluorescence analysis of PS1 expression in methanol acetone fixed IMR-32 cells after transfection with PS1 siRNA and control siRNA**. Plane 1: Non siRNA ransfected IMR-32 Cells; Plane 2: Control siRNA; Plane 3: 50 pmoles PS1siRNA; Plane 4: 75 pmoles PS1 siRNA; Plane 5: 100 pmoles PS1 siRNA. PS1 siRNA and control siRNA transfected IMR-32 cells were probed with PS1 antibody. PS1 siRNA and control siRNA transfected IMR-32 cells were probed with anti PS1 antibody. The signal was revealed using FITC-conjugated secondary antibody and visualized on a fluorescence microscope (o.m. 10X).

In order to confirm the results obtained from western blotting further immunoflourescence was carried out after transfecting IMR-32 cells with the control siRNA and PS1 siRNA in the same concentrations (50 **pmoles**, 75 **pmoles **and 100 **pmoles**) using antibodies against PS1 and Aβ42. As is evident from Figure 4 and 5, the expression levels of PS1and Aβ42 after transfection almost ceased at 75 and 100 **pmole concentration**. Therefore, down regulation of PS1 using PS1 siRNA results in down regulation of Aβ42 as well.

## Discussion

**γ-Secretase activity is responsible for the cleavage of the transmembrane domain of the amyloid precursor protein (APP), releasing the amyloid peptide Aβ and the APP intracellular domain. Aβ is a major component of the amyloid plaques characteristic of Alzheimer's disease**. Presenilins (PS1 and PS2) appear to be essential for γ-secretase cleavage **and form an essential part of the γ-secretase complex**. PS1 knockout markedly inhibits γ-secretase cleavage [19], whereas a PS2 knockout has no apparent effect on γ-secretase activity [20], probably owing to compensation by the more widely expressed PS1. Recent studies have identified PS1 as a part of γ- secretase complex. To test the role of PS1 in Aβ42 production and find out new drug and gene therapy for AD, in the present study IMR-32 cells were transfected with siRNA against PS1gene, and in order to interfere with transcription of PS1 gene, and then determined the levels of PS1 mRNA, PS1 and Aβ42 protein levels. Our data demonstrated that suppression of PS1 gene in IMR-32 cells decreased the production of Aβ42, suggesting that PS1 contributes to γ-secretase activity. The final step of Aβ biogenesis is the processing of CTFγ by γ-secretase within the βAPP transmembrane domain. With the single exception of the Swedish βAPP mutation (K670 to N and M671 to L) all the identified changes linked to early onset familial Alzheimer disease (FAD) affect γ-secretase processing to specifically increase the levels of Aβ42 [21]. To date over 50 FAD linked mutations in the chromosome 14 encoded presenilin 1 (PS1) have been identified, and several more in presenilin 2 (PS2). Previous studies have identified several proteins that bind PS1 but their roles at least partly appear to be related to the function of PS1, other than γ-secretase activity such as regulation of apoptosis and calcium signaling [22]. On the other hand studies on mutant presenilins in transgenic animals have shown increase in the relative level of Aβ42 production [23,24]. The blocking expression of PS1 protein using siRNA results in a loss of γ-secretase activity. In this study, we wanted to know whether suppression of PS1 could regulate γ-secretase activity in IMR-32 cells and regulate Aβ42 production. The cell line was characterized by the increased γ-secretase activity and Aβ42 production in AD [25]. The fact that PS1 is at least partially limiting the Aβ42 production suggests that γ-secretase is indeed a complex including PS1 and the complex activity which can be increased by the expression or reinforcement of any one of its components. AβPP is cleaved by β-secretase to CTFβ, which is subsequently processed by γ-secretase to Aβ40/42 and CTFγ50. A 79-residue fragment between the two cleavage sites has not yet been detected. Multiple studies indicate that γ-secretase is a large multisubunit enzyme [26].

Based on mutagenesis and inhibitor cross-linking studies, it has been suggested that PS1 constitute the catalytically active subunit of γ-secretase. However, PS1 is not sufficient for γ-secretase activity in vitro and multiple subunits have been identified that are essential for generation of the active enzyme. Several of these subunits are apparently limiting for γ-secretase activity in the cell. Since increase in Aβ42 is consistently linked to AD pathogenesis [26], the finding that levels of PS1 can regulate the production of this fragment makes it important candidate for evaluation in AD. The reduction in the relative Aβ42 levels is consistent with the observed inhibition of γ-secretase with a variety of inhibitors [27], which may play an important protective function in the brain. Thus, the reinforcement of γ-secretase activity may be one of the reasons that cause AD. Alternatively, the increase in Aβ42 may be a marker of the reinforcement of γ-secretase activity, which may result in the reinforcement of function of a number of γ-secretase substrates including Notch.

## Conclusion

So in the present study we have shown that siRNA against PS1 down regulates amyloid beta 42 production in IMR32 cells. Taken together the results of the present study suggest that in pathogenic condition we may target PS1 for anti-amyloidogenic therapy in Alzheimer's disease as IMR32 is an AD featured cell line. These results also reconfirm a role of PS1 in γ-secretase activity.

## Abbreviations

Aβ: Amyloid β; AD: Alzheimer disease; APP: amyloid precursor protein; CTF: C-Terminal fragment; FAD: Familial Alzheimer disease; NTF: N-Terminal fragment; PS: presenilin; siRNA: small-interfering RNA.

## Competing interests

We don't have any potential conflicts regarding all financial and material support for this research.

## Authors' contributions

RJLK planned the experimental design, analyzed and interpreted data, and drafted the manuscript. WYW acquired and interpreted data and drafted the manuscript. BKBK analyzed data. KDG planned the experimental design and analyzed and interpreted data. All authors read and approved the final manuscript.
